# Integration of natural and deep artificial cognitive models in medical images: BERT-based NER and relation extraction for electronic medical records

**DOI:** 10.3389/fnins.2023.1266771

**Published:** 2023-09-04

**Authors:** Bo Guo, Huaming Liu, Lei Niu

**Affiliations:** ^1^School of Computer and Information Engineering, Fuyang Normal University, Fuyang, China; ^2^Department of Computing, Faculty of Communication, Visual Art and Computing, Universiti Selangor, Bestari Jaya, Selangor, Malaysia

**Keywords:** medical informatics, decision support system, deep artificial cognitive model, natural language processing, electronic medical record, named entity recognition (NER), BERT model

## Abstract

**Introduction:**

Medical images and signals are important data sources in the medical field, and they contain key information such as patients' physiology, pathology, and genetics. However, due to the complexity and diversity of medical images and signals, resulting in difficulties in medical knowledge acquisition and decision support.

**Methods:**

In order to solve this problem, this paper proposes an end-to-end framework based on BERT for NER and RE tasks in electronic medical records. Our framework first integrates NER and RE tasks into a unified model, adopting an end-to-end processing manner, which removes the limitation and error propagation of multiple independent steps in traditional methods. Second, by pre-training and fine-tuning the BERT model on large-scale electronic medical record data, we enable the model to obtain rich semantic representation capabilities that adapt to the needs of medical fields and tasks. Finally, through multi-task learning, we enable the model to make full use of the correlation and complementarity between NER and RE tasks, and improve the generalization ability and effect of the model on different data sets.

**Results and discussion:**

We conduct experimental evaluation on four electronic medical record datasets, and the model significantly out performs other methods on different datasets in the NER task. In the RE task, the EMLB model also achieved advantages on different data sets, especially in the multi-task learning mode, its performance has been significantly improved, and the ETE and MTL modules performed well in terms of comprehensive precision and recall. Our research provides an innovative solution for medical image and signal data.

## 1. Introduction

Medical imaging and signal processing play an increasingly crucial role as key data sources in the field of medicine. It contains key information such as the patient's physiology, pathology, and genetics (Paredes-Orta et al., 2022). Through these data, medical professionals can obtain patients' physiological information, pathological features, and genetic background, enabling more accurate diagnoses and personalized treatment plans. However, due to the complexity and diversity of medical images and signals, traditional processing methods often struggle to fully unleash the potential of the data, limiting the acquisition of medical knowledge and decision support (Remeseiro and Bolon-Canedo, 2019).

To address this challenge, this paper aims to explore the integration of natural and artificial cognitive systems in medical imaging and signal processing, with a particular focus on applications in medical imaging. Specifically, we will concentrate on electronic medical records (EMRs), an essential form of medical data (Chang et al., 2009). An electronic medical record (EMR) is a digital record of a patient's medical history, including medical records, text, symbols, charts, images, and data, generated during medical activities (Gallegos-Duarte et al., 2020). It replaces traditional paper-based records, storing, managing, transmitting, and reproducing patient medical information in electronic form (Sanchez-Reyes et al., 2020). EMRs typically contain various medical data, such as patient personal information, disease diagnoses, treatment plans, medical orders, medication prescriptions, and results of auxiliary examinations. When NER tasks involve the medical field, they can be roughly divided into disease and symptom recognition, drug entity recognition, medical code and term recognition, etc. The application of these specific NER tasks can enable doctors, researchers, and public health officials to better understand and analyze medicine text data (Wu et al., 2020). Additionally, EMRs serve as a primary corpus for NER, providing crucial data support for applications such as medical decision-making, medical information retrieval, and medical intelligent question-answering. However, the text format of EMRs is often semi-structured or even unstructured (Huang et al., 2021), and it involves numerous medical terms and specialized expressions, posing significant challenges for traditional text processing methods in effectively handling and mining information from EMRs. To overcome these challenges and bring greater value to the medical field, the application of Natural Language Processing (NLP) technology in EMRs has gained significant research attention (Savova et al., 2019).

NER is an NLP technique used to identify entities with specific meanings from text, such as names of people, locations, organizations, dates, times, drugs, etc. In the field of electronic medical records, the goal of NER is to recognize medically relevant entities from structurally diverse EMRs and classify them into predefined categories, such as diseases, symptoms, diagnoses, examinations, and other medical entity types. Through NER, healthcare professionals can efficiently extract critical information from electronic medical records, assist clinical decision-making, and provide more personalized medical services. In addition to NER, when it comes to Relation Extraction (RE) tasks in natural language processing, our objective is to identify semantic relationships between entities in the text (Fu et al., 2019). These entities can be specific objects represented in the text, such as individuals, locations, organizations, drugs, diseases, etc. The purpose of RE is to extract associations between these entities from the text, such as “X treats Y,” “X belongs to Y,” “X is located in Y,” and other relationships. In the medical domain, RE becomes especially crucial to overcome the challenges posed by the complex medical terminologies and specialized expressions commonly found in medical texts.

In recent years, with the widespread application and digital transformation of electronic medical records, NER in electronic medical records has become a highly researched and focused area. This is also attributed to the organization of evaluation competitions, which provide scholars with a platform to exchange and showcase their research achievements. The emergence of these competitions has sparked in-depth research and exploration of NER in electronic medical records by numerous scholars from both domestic and international communities. Through the summarization and synthesis of NER methods in electronic medical records, researchers have compared and evaluated different approaches and technologies, providing essential references and guidance for the advancement of this field (Alfonso-Francia et al., 2022). Among them, Li et al. (2019) summarized the methods based on dictionaries, rules, and machine learning for NER, entity attribute recognition, and entity RE in electronic medical records. Lin et al. (2019), starting from the effectiveness and performance of models, analyzed the current status and challenges of clinical electronic medical record information extraction. Meanwhile, Solares et al. (2020) approached from the perspective of model architectures, presenting an overview and analysis of the strengths and weaknesses of each model type. These research findings and syntheses have not only provided scholars with a clearer understanding of NER in electronic medical records but also propelled the development and innovation in this field. Through the collective efforts of scholars from both domestic and international communities, the techniques and methods for NER in electronic medical records have continuously improved and advanced, providing robust support and assurance for the mining and utilization of medical information.

However, this approach has limited coverage and relies on manually constructed rules and dictionaries, making it challenging to adapt to newly emerging terms and diverse expressions in electronic medical records. Machine learning-based methods, on the other hand, train models to learn entity features, enabling automatic recognition of new entities and diverse expressions.

In this study, due to the excellent performance of BERT pre-trained language model on NER tasks, we adopted an end-to-end framework based on the BERT model, integrating NER and RE tasks into a unified model, avoiding the limitations and error propagation of traditional methods with multiple independent steps.

We also consider the instability of the model training process. Datta et al. (2023) found that DNNs have query prediction instability when performing natural language understanding (NLU) tasks, and proposed a data-centric approach to exploit local stability The method of linear estimation reduces the computational cost and improves the stability of the model. Hidey et al. (2022) found that when retraining the model, using different random seed initializations can lead to model loss and jitter problems, and reduce the number of users by introducing consistency indicators and distillation between predictions in multiple retrainings drain.

After comprehensive consideration, we used the BERT model as the basis for pre-training and pre-trained it on large-scale electronic medical record data to obtain rich semantic representations. Subsequently, we fine-tuned the BERT model to improve the stability of the model, and further trained it on specific NER and RE tasks to adapt to the specific requirements and tasks in the field of electronic medical records. In this process, we employed multitask learning, sharing the underlying representations of the BERT model, enabling the model to leverage the correlations and shared knowledge between different tasks, thereby improving the model's generalization ability and effectiveness. The experimental results demonstrate that our proposed EMLB (End-to-End Multitask Learning BERT) model achieved significant and consistent improvements compared to other baseline models.

Our research work holds significant practical implications for the automation of electronic medical record (EMR) processing (Chelladurai and Pandian, 2022). It can assist medical personnel in rapidly extracting critical information from EMRs, aiding in clinical decision-making and providing more personalized and high-quality medical services. Additionally, it can enhance convenience and safety for patients during medical examinations and treatments. Moreover, our approach provides robust support for the field of medical imaging and signal processing by extracting connections between patients and diseases or treatments from EMRs, offering vital references for the analysis and utilization of medical image and signal data. The integration of natural and artificial cognitive systems in medical imaging and signal processing holds great importance for the acquisition of medical knowledge and decision support. Particularly, in the context of EMR processing and mining, the application of NLP technology provides essential data support for medical intelligent question-answering, information retrieval, and other applications. Through this research, we hope to drive advancements in medical informatics (Tian et al., 2019) and make positive contributions to the development of the medical field.

The contributions of this paper can be summarized in the following three aspects:

1. This paper uses the BERT model as the main word embedding representation method, and applies the MC-BERT (Medical Transformer-based bidirectional encoding representation) pre-training model in the electronic medical record text. By using the BERT model, it is possible to obtain rich semantic representation capabilities and improve the recognition accuracy of named entities in electronic medical record texts.
2. This paper proposes an end-to-end framework to integrate the electronic medical record NER and RE tasks into a unified model. Traditional methods often employ multiple independent steps, limiting the performance and effectiveness of the model. Adopting an end-to-end framework can avoid these limitations and error propagation, and improve the performance of the overall system.
3. This paper adopts the idea of multi-task learning, by sharing the underlying representation of the BERT model, and fine-tuning on specific NER and relationship extraction tasks at the same time. This method can make full use of the correlation and shared knowledge between different tasks, and improve the generalization ability and effect of the model.

The logical structure of this paper is as follows: In Section 2, the related work is reviewed, focusing on the research progress in information extraction from electronic medical records. In Section 3, our methods are presented in detail, including the introduction of the BERT model, the end-to-end framework, and the concept of multi-task learning, along with its specific implementation. The experimental section, Section 4, provides a comprehensive overview of the experimental settings, encompassing the datasets, evaluation metrics, and comparative methods used. Additionally, it compares the experimental results obtained from different approaches. In Section 5, the discussion section, we analyze the advantages of our model, while also pointing out limitations and proposing future improvements. Lastly, Section 6, the conclusion, summarizes the contributions and significance of the paper, and also outlines potential directions for future research and related work.

## 2. Related work

With the advancement of medical information technology, a vast amount of medical images and their corresponding electronic medical record (EMR) texts are being generated. Extracting valuable clinical information from this multimodal medical data has become a crucial focus in medical artificial intelligence research. In this section, we will review related works and explore the latest progress in the integration of natural and artificial cognitive systems in medical image and signal processing.

In previous research, methods based on rules, statistical machine learning, and deep learning have achieved certain accomplishments. However, when faced with unstructured EMR texts, these methods still encounter challenges, such as word sense disambiguation, entity boundary recognition, and entity relationship extraction. In a medical research article written by Dr. Xue, there is such a sentence “Our's study revealed a strong link between diabetes and cardiovascular complications.” Extracting the relationship between “diabetes” and “cardiovascular complications” is an entity relationship extraction task. These relationships are often complex and require understanding the context and semantics of the text (Xue et al., 2019). Zhang's article mentions that in the patient's EMR, the sentence “patient heart attack” needs to be parsed to extract the medical condition “heart attack” and the context of the patient. Processing unstructured EMR text requires models to understand medical terminology, context, and relationships, which can be complex due to variations in wording and grammar (Zhang et al., 2019).

Among the various techniques for NER, four main approaches are prominent: (1) rule-based methods, (2) dictionary-based methods, (3) machine learning-based methods, and (4) deep learning-based methods. In the study of RE, “relation” refers to the semantic association between two entities present in the text. The goal of RE is to extract and classify the relationships between these entities, providing more comprehensive information for medical information processing and aiding healthcare professionals in better understanding the connections and interactions between entities.

In recent years, deep learning based methods have achieved remarkable results in RE. Especially methods based on pre-trained models, such as BERT, have made important breakthroughs in RE tasks in the medical field by learning rich semantic information. Researchers can use pre-trained models such as BERT to extract the relationship features between entities, so as to achieve more accurate and efficient relationship extraction.

The standard BERT model, fundamentally built upon the Transformer architecture, leverages bidirectional contextual information during its training to encapsulate a broader context. Its primary pre-training objective involves learning the representation of each position in a sentence via masked language modeling (MLM) (Wang and Cho, 2019). This approach facilitates the model's comprehension of word interrelationships. While architectures akin to standard BERT predominantly assign weights based on the significance of each position in the input sequence, introducing context during sequence processing, their purposes vary. Although both the standard attention module and BERT incorporate attention mechanisms, they serve different ends. The former focuses on sequence processing tasks, whereas BERT seeks to master universal language representations through pre-training, which can later be fine-tuned for diverse downstream tasks.

Feature engineering plays a crucial role in both electronic medical record NER and RE, as extracting rich and effective features can significantly enhance model performance. Due to the specific nature of electronic medical records, the design of feature engineering often relies on the characteristics inherent to these records. Additionally, RE tasks require attention to the contextual information between entities to better understand their associations. Electronic medical records, being vital textual data in the medical field, exhibit several prominent characteristics. Firstly, they contain abundant medical terminologies and specialized vocabulary, such as diseases, symptoms, and medications. These terms and vocabulary are crucial for entity recognition. Secondly, the textual structure of electronic medical records typically includes sections like medical record titles, summaries, diagnoses, treatments, etc., which can be utilized to assist entity recognition tasks. Furthermore, electronic medical records often include entity information such as time, location, and names, which also need to be accurately recognized and classified. One common method of feature engineering is rule-based, as seen in Cui et al. (2019), where a large-scale regular expression is constructed to establish medical rules for identifying named entities like drugs and dosages in clinical records. Another frequently used approach is based on dictionaries. Researchers build medical dictionaries and term lists to match predefined entity terms with the electronic medical record text. Through this matching process, known entities can be effectively identified, thus improving recognition accuracy. For instance, Ji et al. (2019) proposed a dictionary-based NER method for electronic health records.

In the early research of electronic medical record NER, traditional machine learning methods were relied upon, and some notable research achievements were obtained. For example, Souza et al. (2019) combined conditional random field (CRF) models with part-of-speech tagging and other handcrafted features to achieve good performance in disease NER. This work demonstrated that the CRF model can effectively integrate textual feature information, significantly improving the performance of medical text entity recognition based on traditional machine learning methods. However, this CRF method that heavily relies on manual feature engineering also faces the limitation of requiring a large amount of annotated training data. With the rise of deep learning, feature engineering methods based on deep learning have also received widespread attention. Among them, methods based on pre-trained models have become a research hotspot. BERT, as a commonly used pre-trained model, has achieved significant results in electronic medical record NER. Researchers can utilize the BERT model to extract features at the character, word, and sentence levels, capturing rich semantic information. For instance, Roy and Pan (2021) described in the article that the BERT model was used to implement medical entity RE and achieved state-of-the-art results on medical relation datasets. This shows that utilizing BERT pre-training can produce better results than traditional feature engineering methods. Ji et al. (2020) pointed out that the combination of LSTM-CRF network with symptom-based character-level features improves the recognition performance of medical terms. Additionally, Lee et al. (2019) pointed out that the BERT + Bi-LSTM + Attention fusion model was proposed, utilizing BERT-transformed medical text as feature representations and employing Bi-LSTM and attention layers to accomplish medical record text extraction and classification tasks.

In medical NER research, model fusion methods remain mainstream and are often combined with manual rules. Common model fusion strategies include:

- (1) Stacking: Abstracting the medical NER task into several consecutive sub-steps and employing different model methods for each sub-step to improve overall recognition accuracy. For instance, in Dai et al. (2019), CRF was replaced with BiLSTM at the end of the SoftMax layer, and the two were combined to fully learn the information between annotations, resulting in improved model performance.
- (2) Voting: Voting on the prediction results of different models and filtering high-confidence results by setting thresholds. This method can effectively filter false-positive results and enhance the confidence of prediction outcomes. For example, in Gao et al. (2021), a dual-threshold voting approach was utilized, where a high threshold was set to obtain high-confidence results, and the resulting vocabulary guided the low-threshold voting process to improve model performance.
- (3) Hybrid: Hybrid fusion includes method fusion and model fusion. Method fusion combines machine learning methods with rule-based methods to correct model prediction results and practical biases. Model fusion, on the other hand, improves accuracy by combining the prediction results of multiple models. For example, in Almeida and Xexéo (2019), a fusion of models based on character features and word features was employed, leading to enhanced model performance. In Nagrani et al. (2021), three fusion models were quadratically combined, and rules and dictionaries were integrated into the model, significantly improving the final recognition of medical named entities.

Combining the integration of natural and deep artificial cognitive models in medical image and signal processing, especially the BERT-based method for NER and relationship extraction in electronic medical records, the extracted text entities will be used as prior knowledge of medical images to guide visual models such as image segmentation and classification to better process medical images. The ultimate goal is to realize the integration of natural language processing and computer vision technology, build a medical artificial intelligence system that imitates the human cognitive process, and improve the ability of comprehensive analysis and understanding of medical record images. By making full use of the advantages of natural and artificial cognitive systems, we are expected to achieve more accurate, efficient and personalized medical data processing and decision support, and make positive contributions to the improvement of patients' health and medical system.

## 3. Methodology

Our approach is based on the BERT model for NER and RE tasks in electronic medical records. First, we employ an end-to-end framework, directly starting from raw input data. The BERT model combines components such as self-attention, feed-forward neural networks, and layer normalization to achieve deep representation learning from input data. Then, a multi-task learning strategy is adopted to combine NER and RE tasks to share model representation capabilities and improve comprehensive performance. Next, task-specific layers are added for the two tasks, predicting entity and relation categories through linear transformation and non-linear activation. During training, we simultaneously optimize the loss functions for both tasks to minimize error. With this approach, we are able to simultaneously achieve efficient and accurate NER and RE in electronic medical records.

### 3.1. BERT model

**Figure 1:**
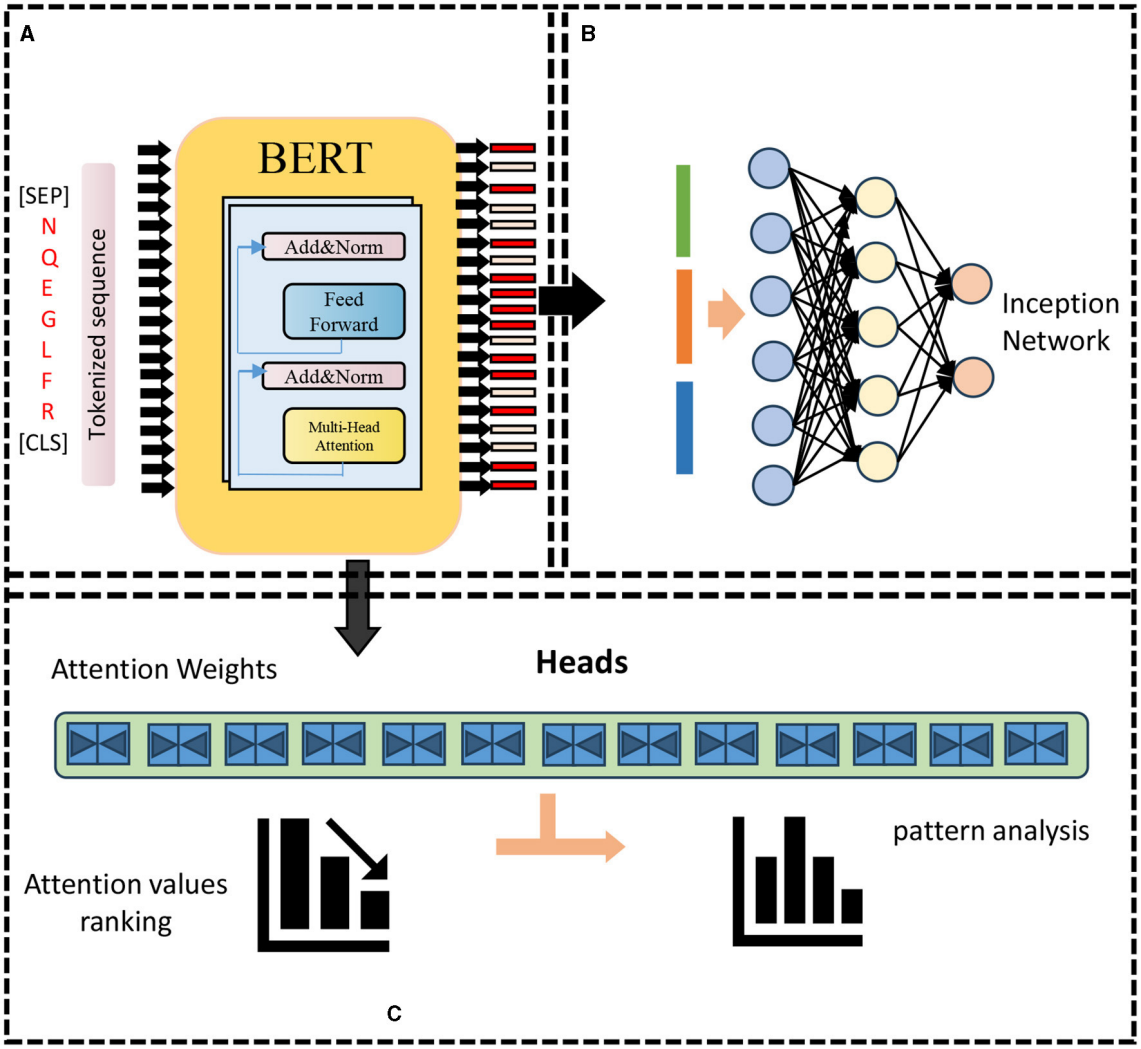
BERT model framework. **(A)** Pre-training: features encoding. **(B)** Re-training: prediction. **(C)** Attention weights analysis.

The BERT model is a two-way autoencoder model based on the Transformer encoder structure (Acheampong et al., 2021). It is stacked by *L*-layer Transformer encoders. Each layer of Transformer encoders consists of a multi-head self-attention (sublayer and a feedforward neural network sublayer composition, there are residual connections and layer normalization between the two sub-layers). The input of the BERT model is a matrix of shape (*n, d*) where *n* is the length of the input sequence and *d* is the embedding dimension. The BERT model The output is a matrix of shape (*n, h*), where *h* is the size of the hidden layer. The parameter amount of the BERT model is *O*(*Lnh*^2^+*Lnhd*), where *L* is the number of layers, *n* is the sequence length, and *h* is the size of the hidden layer. *d* is the embedding dimension. As shown in Figure 1.

We take the *l*-th layer Transformer encoder as an example, given the input matrix *X*^(*l*)^ ∈ *R*^*n*×*h*^, where *n* is the sequence length, *h* is the size of the hidden layer, the output matrix *Y*^(*l*)^ ∈ of the l-th layer Transformer encoder *R*^*n*×*h*^ can be expressed as:


(1) $$\begin{array}{l} Y^{l}=FFN\left(LN\left(X^{l}+MHA\left(X_{1}^{l},...,X_{N}^{l}\right)\right)\right) \end{array}$$

Among them, FFN represents the feed-forward neural network sublayer, *LN* represents the layer normalization operation, and MHA represents the multi-head self-attention sublayer. Below we introduce the specific formulas of these three operations.

• Feedforward neural network sublayer: The feedforward neural network sublayer is a two-layer fully connected network that performs a non-linear transformation on each row in the input matrix (Vucetic et al., 2022). Given an input matrix *Z* ∈ *R*^*n*×*h*^, the output matrix *W* ∈ *R*^*n*×*h*^ of the feedforward neural network sublayer can be expressed as:


(2) $$\begin{array}{l} W=\text{ReLU}\left(Z\in R^{n\times h}\right)W_{1}+b_{1} \end{array}$$

where ReLU represents the linear rectification unit activation function, $W_{1}\in R^{h\times d}$ is a learnable weight matrice, $b_{1}\in R^{d}$ is a learnable bias vector, *d* is the size of the middle layer of the feedforward neural network sublayer.

• Layer normalization operation: Layer normalization operation is a method of normalizing each row in the input matrix, which can improve the convergence speed and generalization ability of the model (Shi et al., 2022). Given an input matrix *Z* ∈ *R*^*n*×*h*^, the output matrix *U* ∈ *R*^*n*×*h*^ of the layer normalization operation can be expressed as:


(3) $$\begin{array}{l} U=G⊙\frac{Z-\text{mean}\left(Z\right)}{\sqrt{\text{var}\left(Z\right)+\epsilon }}+B \end{array}$$

where ⊙ represents the Hadamard product (element-wise multiplication), mean(*Z*) ∈ *R*^*h*^ and var(*Z*) ∈ *R*^*h*^ represent the mean and variance of each column of the input matrix, respectively, and ∈ is a small positive number used to prevent division With zero error, *G* ∈ *R*^*h*^ and *B* ∈ *R*^*h*^ are two learnable scaling and translation vectors.

**Figure 2:**
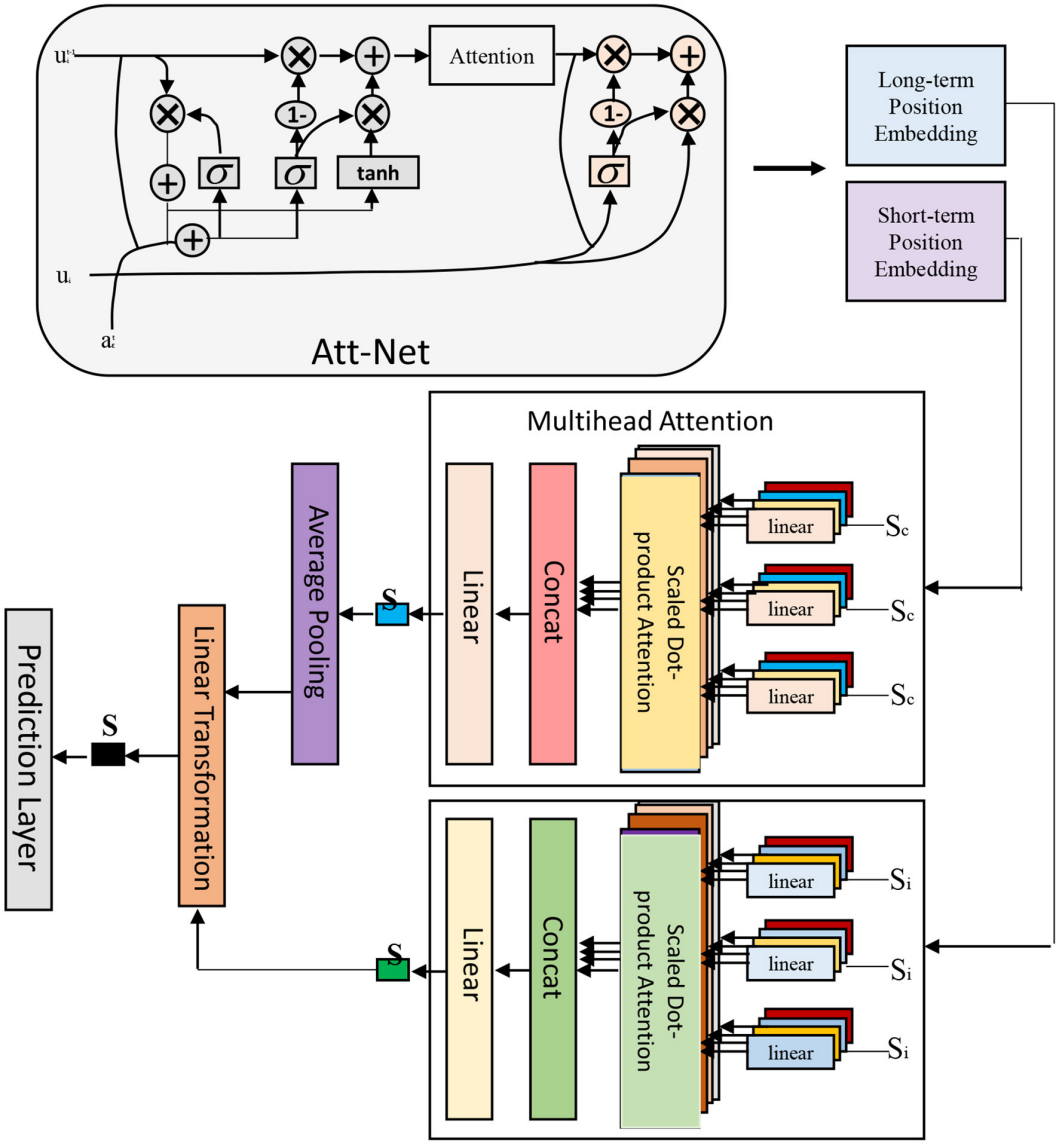
Multi-head attention framework.

• Multi-head self-attention sublayer: The multi-head self-attention sublayer is a method that uses the self-attention mechanism to capture the dependencies between different positions in the input sequence, which can simultaneously consider the bidirectional contextual information (Wu et al., 2019). As shown in Figure 2.

Given an input matrix *Z* ∈ *R*^*n*×*h*^, the output matrix *V* ∈ *R*^*n*×*h*^ of the multi-head self-attention sublayer can be expressed as:


(4) $$\begin{array}{l} V=\text{Concat}\left(\text{hea}{\text{d}}_{1},\text{hea}{\text{d}}_{2},\ldots ,\text{hea}{\text{d}}_{m}\right)W_{0} \end{array}$$

where Concat represents the concatenation operation, m is the number of heads in the multi-head self-attention sublayer, *head*_*i*_ represents the output matrix of the *i*-th self-attention head, and $W_{0}\in R^{h\times h}$ is a learnable weight matrix. The output matrix *head*_*i*_ of each self-attention head can be expressed as:


(5) $$\begin{array}{l} \text{hea}{\text{d}}_{i}=\text{Attention}\left(ZW_{Q}^{\left(i\right)},\;ZW_{K}^{\left(i\right)},\;ZW_{V}^{\left(i\right)}\right) \end{array}$$

Where Attention represents the scaled dot-product attention function,$W_{Q}^{\left(i\right)}\in R^{h\times d_{k}}$,$W_{K}^{\left(i\right)}\in R^{h\times d_{k}}$ and $W_{V}^{\left(i\right)}\in R^{h\times d_{k}}$ is Three learnable weight matrices, *d*_*k*_ and *d*_*v*_ are the dimensions of the key and value of each self-attention head, respectively. The scaled dot product attention function can be expressed as:


(6) $$\begin{array}{l} \text{Attention}\left(Q,K,V\right)=\text{Softmax}\left(\frac{QK^{T}}{\sqrt{d_{k}}}\right)V \end{array}$$

Among them, Softmax represents the softmax function, which can normalize each row of the input matrix into a probability distribution,$Q\in R^{n\times d_{k}}$, $K\in R^{n\times d_{k}}$ and $V\in R^{n\times d_{v}}$ respectively, represent query, key and the value matrix, *d*_*k*_ and *d*_*v*_ represent the dimensions of the key and value, respectively. The role of the zoom dot product attention function is to calculate the weighted sum of the value according to the similarity between the query and the key, where the scaling factor $\frac{1}{\sqrt{d_{k}}}$ is to prevent the gradient of the softmax function from disappearing due to the excessive dot product result.

### 3.2. End-to-end framework

**Figure 3:**
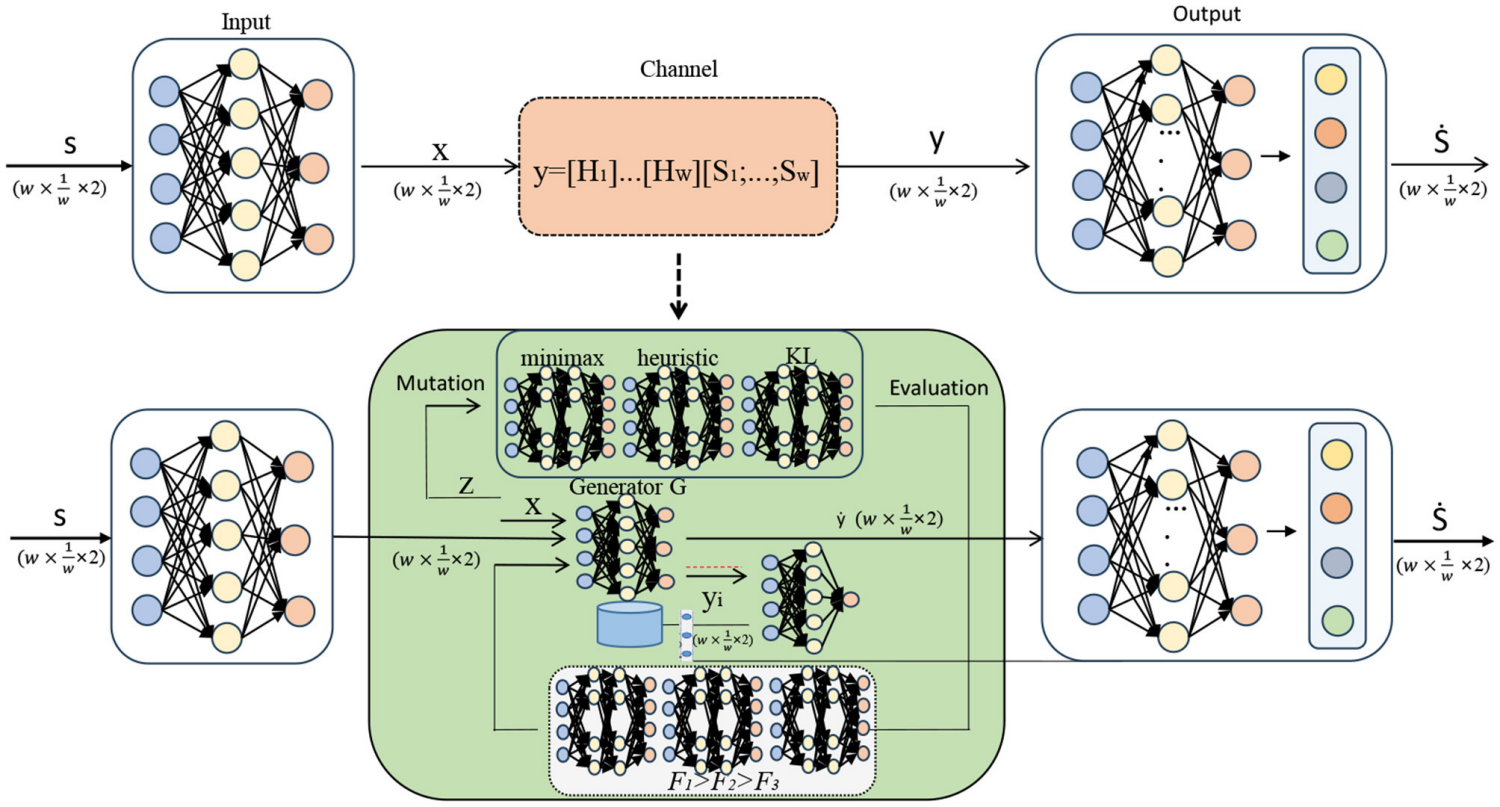
End-to-end framework.

In BERT-based NER and RE for electronic medical records, an end-to-end framework is adopted, which can directly process from raw input to final output without explicit feature engineering or human-designed rules. As shown in Figure 3.

It consists of two main parts: entity labeling part and relation classification part (Feng et al., 2019).

• Entity labeling part: The entity labeling part is responsible for identifying different types of entities, such as diseases, drugs, and inspections, from electronic medical record texts. We employ a CRF based sequence annotation method that can leverage contextual information and constraints between labels to improve entity recognition accuracy. Given the output matrix *Y*^(*L*)^ ∈ *R*^*n*×*c*^ of the pre-training layer, the output of the entity labeling part is a matrix *P* ∈ *R*^*n*×*c*^ of shape (*n, c*), where *c* is the number of entity categories. The output matrix P of the entity labeling part can be expressed as:


(7) $$\begin{array}{l} P=\text{CRF}\left(Y^{\left(L\right)}W_{E}+b_{E}\right) \end{array}$$

Where CRF represents the conditional random field layer, $W_{E}\in R^{h\times c}$ and $b_{E}\in R^{c}$ are two learnable weight matrices and bias vectors. The function of the conditional random field layer is to calculate the probability of each category at each position according to the input sequence and the transition matrix, and find the optimal label sequence through the Viterbi Algorithm.

• Relation classification part: The relation classification part is responsible for extracting different types of relations from electronic medical record texts, such as drug-dose, examination-result, etc. We employ a bilinear attention-based approach for relation classification, which can leverage both semantic similarity and syntactic dependencies between entity pairs to improve the accuracy of RE. Given the output matrix *Y*^(*L*)^ ∈ *R*^*n*×*h*^ of the pre-training layer and the output matrix *P* ∈ *R*^*n*×*c*^ of the entity labeling part, the output of the relation classification part is a matrix *Q* ∈ ℝ^*m*×*r*^ of shape (*m, r*), where m is the number of entity pairs and r is the number of relationship categories. The output matrix *Q* of the relation classification part can be expressed as:


(8) $$\begin{array}{l} Q=\mathrm{BA}\left(\text{Gather}\left(Y^{\left(L\right)},P\right)\right)W_{R}+b_{R} \end{array}$$

Among them, *BA* means bilinear attention layer, Gather means to collect the vector corresponding to the position of the entity in the output of the pre-training layer according to the entity label, $W_{R}\in R^{2h\times r}$ and $b_{R}\in R^{r}$ are two learnable weight matrices and bias vectors. The role of the bilinear attention layer is to calculate the probability of each category on each entity pair according to the bilinear similarity between entity pairs, and find the optimal relationship category through a threshold or greedy algorithm.

### 3.3. The idea of multi-task learning

**Figure 4:**
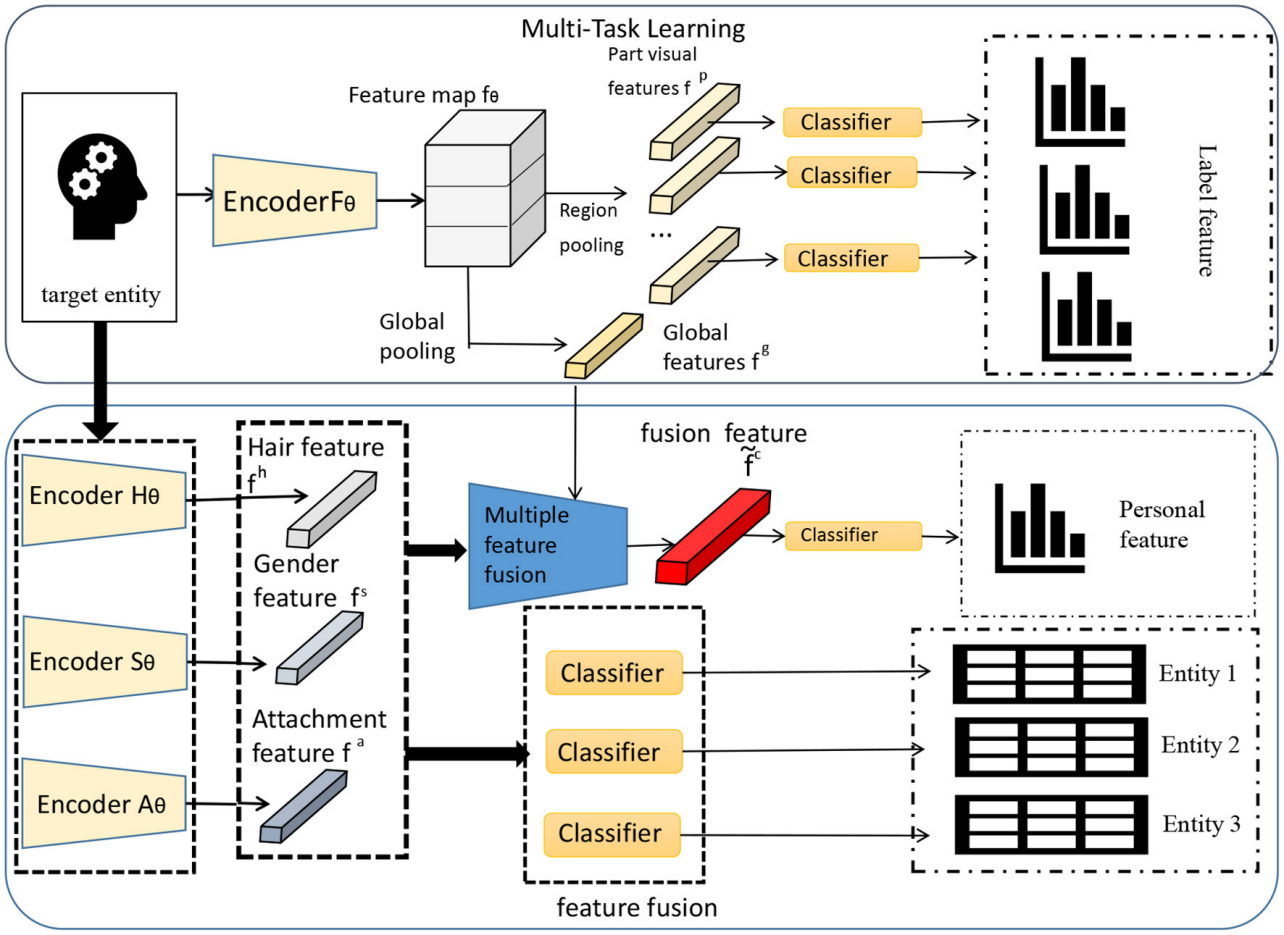
Multi-task learning framework.

In the BERT-based NER and relationship extraction tasks of electronic medical records, the idea of multi-task learning is adopted. This idea can train multiple related tasks at the same time and share the representation ability of the model, thereby improving the performance and generalization ability of the model. As shown in Figure 4.

Multi-task learning can process multiple tasks simultaneously in the same model, bundle different tasks together for training, and share the underlying feature extraction process (Du et al., 2020). This sharing can enable the model to transfer the knowledge and features learned from one task to other tasks, thereby improving the overall performance.

In multi-task learning, we use the shared BERT model as the base model, and then add two separate task-specific layers on top of the model for NER and RE, respectively.

• NER layer: For the NER task, we add a NER layer on top of the BERT model, which consists of a fully connected layer and a softmax classifier. The fully connected layer performs linear transformation on the output of BERT, and performs non-linear mapping through the activation function, and then uses the softmax classifier to predict the entity category.


(9) $$\begin{array}{l} \text{NER}\left(Q\right)=\text{softmax}\left(W_{\text{ner}}\cdot Q+b_{\text{ner}}\right) \end{array}$$

Among them, *NER*(*Q*) represents the prediction result of NER, *h*_*Q*_ is the output vector of the BERT model, *W*_*ner*_ and *b*_*ner*_ are the parameters of the NER layer.

• RE Layer: For the RE task, we add a RE layer on top of the BERT model, which also consists of a fully connected layer and a softmax classifier. The fully connected layer linearly transforms the output of BERT, and performs non-linear mapping through the activation function, and then uses the softmax classifier to predict the relationship category.


(10) $$\begin{array}{l} \text{RE}\left(P\right)=\text{softmax}\left(W_{\text{re}}\cdot h_{P}+b_{\text{re}}\right) \end{array}$$

Among them, *RE*(*P*) represents the prediction result of, *h*_*P*_ is the output vector of BERT model, *W*_*re*_ and *b*_*re*_ are the parameters of layer. During training, we can jointly train the model by minimizing the loss function for both tasks:


(11) $$\begin{array}{l} \text{Loss}=\text{Lossner}+\text{Lossre} \end{array}$$

Among them, *Loss*_*ner*_represents the loss function of the NER task, and *Loss*_*re*_ represents the loss function of the task. By optimizing this multi-task loss function, we can train two tasks simultaneously and enable the model to achieve good performance on NER and tasks.

In order to show the implementation process of the algorithm in this paper more clearly, we provide the pseudocode Algorithm 1, which includes the input parameters of the algorithm, variable definitions, flow control statements, and output results.

**Algorithm 1:** MTL-BERT training.

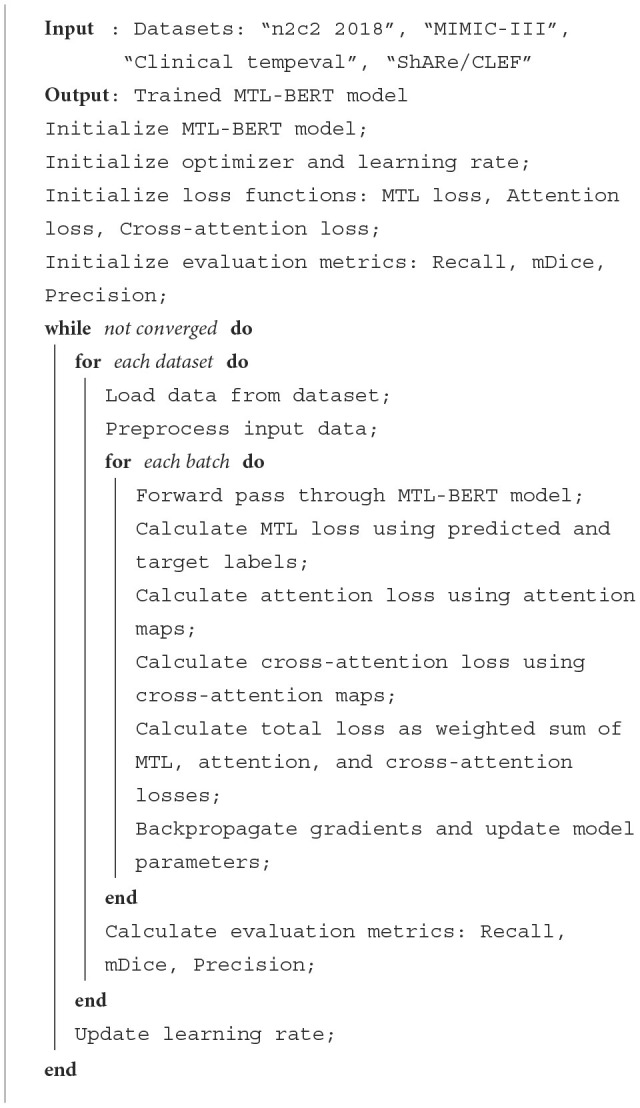

## 4. Experiment

**Figure 5:**
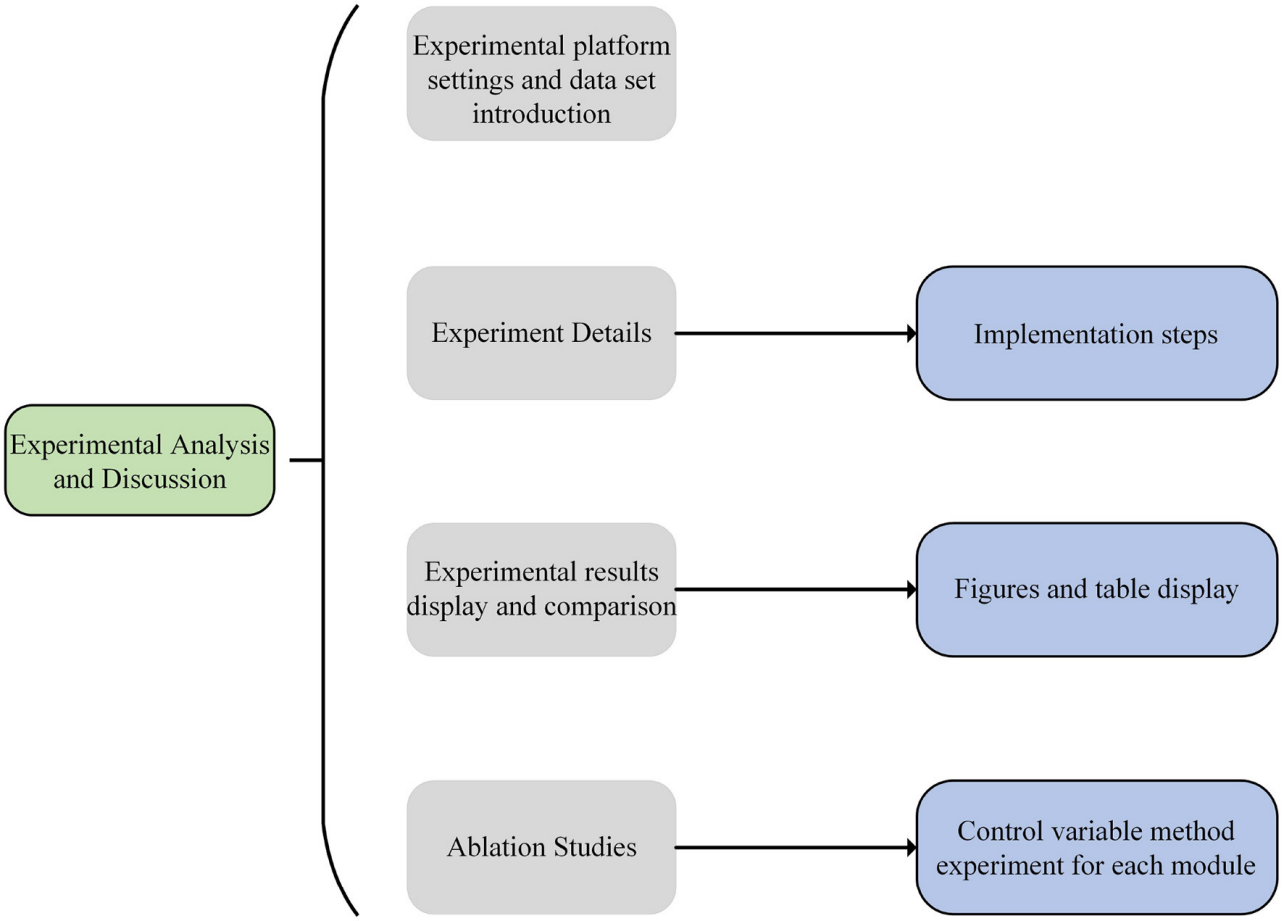
Experiment flow chart.

The experimental process of this paper is shown in Figure 5.

### 4.1. Experimental environment

- Hardware environment
- Our experiments are done on a GPU server with Intel Xeon E5-2690 v4 @ 2.60 GHz CPU, equipped with 512 GB RAM and 8 Nvidia Tesla P100 16 GB GPUs.
- Software environment
- In this study, we used Python and PyTorch to implement BERT-based NER and relationship extraction in electronic medical records under an end-to-end framework. We used the PyTorch-Transformers library, pre-trained model weights, using scripts and transformation tools. We use the BERT model in this library as the basis for pre-training, and obtain rich semantic representation capabilities by pre-training large-scale electronic medical record data.

### 4.2. Experimental data

- n2c2 2018 dataset
- This data set is a data set of a clinical natural language processing challenge organized by the n2c2 project of Harvard Medical School. It contains 296 electronic medical records from the Mayo Clinic in the United States, covering entity types such as drugs, doses, indications, and adverse reactions, as well as relationship types such as drug-dose, drug-indication, and drug-adverse reactions. This dataset is divided into two subtasks: subtask 1 is to extract patient cohorts meeting the inclusion and exclusion criteria of clinical trials from electronic medical records; subtask 2 is to extract drug-related entities and relationships from electronic medical records. This dataset can be used to evaluate the performance of clinical text mining systems in entity recognition and, as well as its application value in clinical trial cohort selection.
- MIMIC-III dataset
- This dataset is a large-scale, diverse, and public critical care database jointly developed by MIT and Beth Israel Deaconess Medical Center. It contains more than 60,000 medical records from more than 40,000 patients, covering information such as vital signs, medication, laboratory tests, doctor's observations and records, fluid balance, surgical codes, diagnostic codes, imaging reports, length of hospital stay, and survival data. This dataset can be used to support various clinical text mining applications such as academic and industrial research, quality improvement programs, higher education courses, etc.
- Clinical tempeval dataset
- This dataset is a dataset of clinical NLP tasks organized by SemEval. It contains 300 de-identified clinical notes selected from the MIMIC-II database, covering clinical events (such as symptoms, diagnosis, treatment, etc.), temporal expressions (such as date, duration, etc.), and the relationship between events and time (such as inclusion, overlap, etc.). This dataset can be used to evaluate the performance of clinical text mining systems in temporal information processing and its application value in constructing clinical timelines.
- ShARe/CLEF dataset
- This dataset is used by the CLEF eHealth information extraction sharing task. It contains 299 de-identified clinical notes selected from the MIMIC-II database, covering death certificate medical concepts (such as anatomical sites, diseases and injuries, etc.) and other annotations. This dataset can be used to evaluate the performance of clinical text mining systems in medical concept recognition and its application value in cause of death analysis.

### 4.3. Evaluation index

In order to evaluate the performance of the NER system, we usually use the following three indicators: precision rate, recall rate and F1 value (Kim et al., 2019). Below are the definitions and formulas for these three metrics, as well as what they mean and what they do.

- Precision: The precision is the ratio of the number of entities correctly recognized as a certain entity category to the total number of recognized entities of that category from the recognition results. It reflects the ability of the model to correctly predict positive samples (i.e., real entities). The higher the accuracy rate, the less likely the model is to generate false positives (i.e., identify non-entities as entities). The formula for accuracy is:
  (12) $$\begin{array}{l} Precision=\frac{TP}{TP+FP} \end{array}$$
- Among them, *TP* stands for True Positive (True Positive), that is, the number of correctly identified entities; *FP* stands for False Positive (False Positive), that is, the number of non-entities incorrectly recognized.
- Recall rate (Recall): The recall rate is the ratio of the number of entities of a certain category correctly identified by the model to the total number of entities of the category (including missed labels). It reflects the ability of the model to find all positive samples. The higher the recall rate, the less likely it is for the model to miss real entities. The formula for the recall rate is:
  (13) $$\begin{array}{l} Recall=\frac{TP}{TP+FN} \end{array}$$
- Among them, *FN* represents False Negative (False Negative), that is, the number of real entities that are missed.
- F1 value (F1-score): F1 value is the harmonic mean of precision and recall. It comprehensively reflects the performance of the model in terms of precision and recall. The higher the F1 value, the better the model can balance precision and recall, avoiding biasing toward one aspect at the expense of the other. The formula for the F1 value is:
  (14) $$\begin{array}{l} F1=\frac{2\times Precision\times Recall}{Precision+Recall} \end{array}$$

Together, these three metrics evaluate the classification accuracy and completeness of the model. A high accuracy rate indicates that the model has a low misjudgment rate for samples predicted as positive examples, and a high recall rate indicates that the model can capture more positive examples. F1-Score combines these two indicators to provide a balanced evaluation index for comprehensively evaluating the performance of the model.

### 4.4. Experimental comparison and analysis

In order to comprehensively evaluate the effectiveness of the proposed method, we conducted evaluations on two tasks: NER and RE. The NER task aims to identify entity categories in the text, while the RE task involves extracting relationships between entities. Both of these tasks are of great significance for deep parsing of Electronic Medical Records (EMRs).

For the experiments, we used publicly available EMR datasets, namely n2c2 2018, MIMIC-III, Clinical tempeval, and ShARe/CLEF. We divide the dataset into 80% training set and 20% test set according to the 80-20 division rule. These datasets were professionally annotated with entity labels and entity relations. We constructed end-to-end NER and RE tasks based on these datasets and trained and tested our proposed EMLB model.

Specifically, the NER task involves classifying entities in the text, for example, labeling “heart disease” as a “disease” type. The RE task, on the other hand, requires determining the relationship between two entities, for example, “amoxicillin—causes—rash” represents a “drug-side effect” relationship.

Under the same experimental settings, we compared the performance of our EMLB model with the methods proposed by Nasar et al. (2021), Fabregat et al. (2023), Govindarajan et al. (2023), Ke et al. (2023), Laursen et al. (2023), Tang et al. (2023). After conducting multiple independent experiments, we used the average F1 score as the evaluation metric. F1 score combines precision and recall, so it can more comprehensively measure the performance of the model. The experimental results demonstrate that our EMLB method outperforms other comparison methods in both tasks, achieving the best performance. Below, we provide a detailed description of the experimental results for both the NER and tasks.

From the results in Table 1, it is evident that our proposed EMLB model outperforms other baseline models in terms of precision, recall, and F1 score for the NER task on the n2c2 2018 and MIMIC_III datasets. Specifically, in the tests on the n2c2 2018 dataset, the EMLB model achieved a precision of 94.33%, a recall of 93.57%, and an F1 score of 93.95%. These metrics surpassed the performance of other models and experiments by Fabregat et al., Nasar et al., and others. The EMLB model's F1 score improved by 2.62 percentage points compared to Fabregat et al.'s experimental method and 15 percentage points compared to Nasar et al.'s experimental results. On the MIMIC-III dataset, the EMLB model achieved precision, recall, and F1 score of 91.61, 91.07, and 91.34%, respectively, which also outperformed the comparison models, demonstrating better identification performance and validating the effectiveness of EMLB in the domain of medical NER.

**Table 1:** Comprehensive evaluation based on precision, recall, and F1 score: different approaches on the n2c2 2018 and MIMIC-III datasets for NER tasks.

**Method**	**Dataset (for NER tasks)**					
	**n2c2 2018 (Henry et al.**, **2020****)**			**MIMIC-III (Wang et al.**, **2020****)**		
	**Precision/%**	**Recall/%**	**F1-score/%**	**Precision/%**	**Recall/%**	**F1-score/%**
Fabregat et al. (2023)	88.32	90.17	91.33	83.98	84.81	84.39
Nasar et al. (2021)	79.78	78.13	78.95	89.82	87.22	88.50
Laursen et al. (2023)	85.86	86.69	86.27	75.47	77.34	76.39
Govindarajan et al. (2023)	82.69	81.97	82.24	87.18	87.81	87.49
Tang et al. (2023)	89.88	89.41	89.64	80.12	81.38	80.75
Ke et al. (2023)	91.16	90.08	90.62	85.36	87.59	86.46
**Ours**	**94.33**	**93.57**	**93.95**	**91.61**	**91.07**	**91.34**

**Figure 6:**
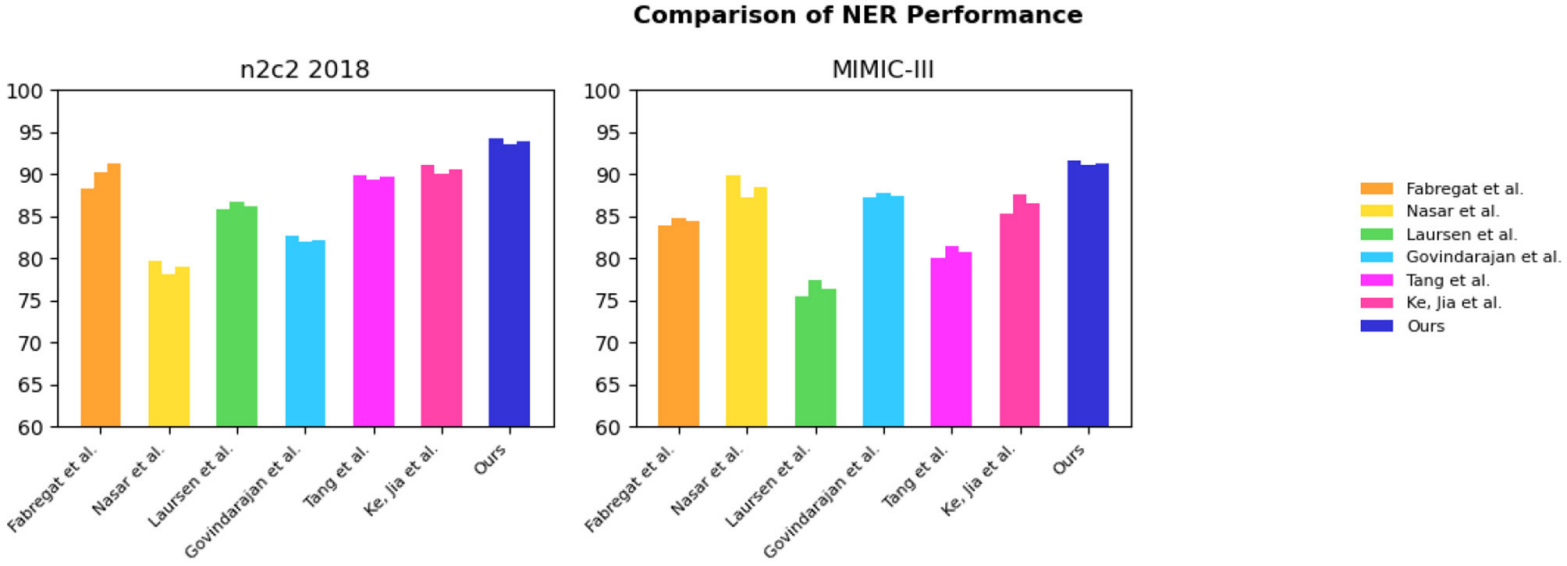
Comparative visualization of NER tasks for n2c2 2018 and MIMIC-III datasets.

To visually compare the results in Table 1, we have presented them in Figure 6.

In Table 2, we show the performance of the model on the n2c2 2018 dataset and the MIMIC-III dataset in terms of the RE task, where the test set is a sample extracted from 20% of the dataset. It can be observed that the EMLB method also outperforms all other comparison methods in the RE task. Specifically, on the n2c2 2018 dataset, the EMLB model achieved an F1 score of 93.97%, which is 2.41% higher than the experimental method proposed by Tang et al. Additionally, the precision and recall of EMLB improved by 1.39 and 3.4%, respectively, compared to Tang et al.'s method. On the MIMIC-III dataset, the EMLB method achieved an F1 score of 93.89%, which is 6.39% higher than the experimental method proposed by Govindarajan et al. The EMLB model demonstrates superior experimental results in the task, indicating the powerful capabilities of pre-trained language models. Through the integration of end-to-end learning and multi-task learning, the EMLB model effectively shared BERT representations for both tasks, leading to the best results in.

**Table 2:** Comprehensive evaluation based on precision, recall, and F1 score: different approaches on the n2c2 2018 and MIMIC-III datasets for RE tasks.

**Method**	**Dataset (for RE tasks)**					
	**n2c2 2018 (Henry et al.**, **2020****)**			**MIMIC-III (Wang et al.**, **2020****)**		
	**Precision/%**	**Recall/%**	**F1-score/%**	**Precision/%**	**Recall/%**	**F1-score/%**
Fabregat et al. (2023)	84.23	85.54	84.88	91.32	88.95	90.12
Nasar et al. (2021)	81.88	82.46	82.17	93.09	91.07	92.07
Laursen et al. (2023)	90.59	86.67	88.59	87.77	87.61	87.69
Govindarajan et al. (2023)	79.36	81.35	80.34	82.82	80.52	81.65
Tang et al. (2023)	76.03	77.15	76.59	84.55	86.18	85.36
Ke et al. (2023)	92.77	90.39	91.56	88.68	86.35	87.50
**Ours**	**94.16**	**93.79**	**93.97**	**94.84**	**92.95**	**93.89**

**Figure 7:**
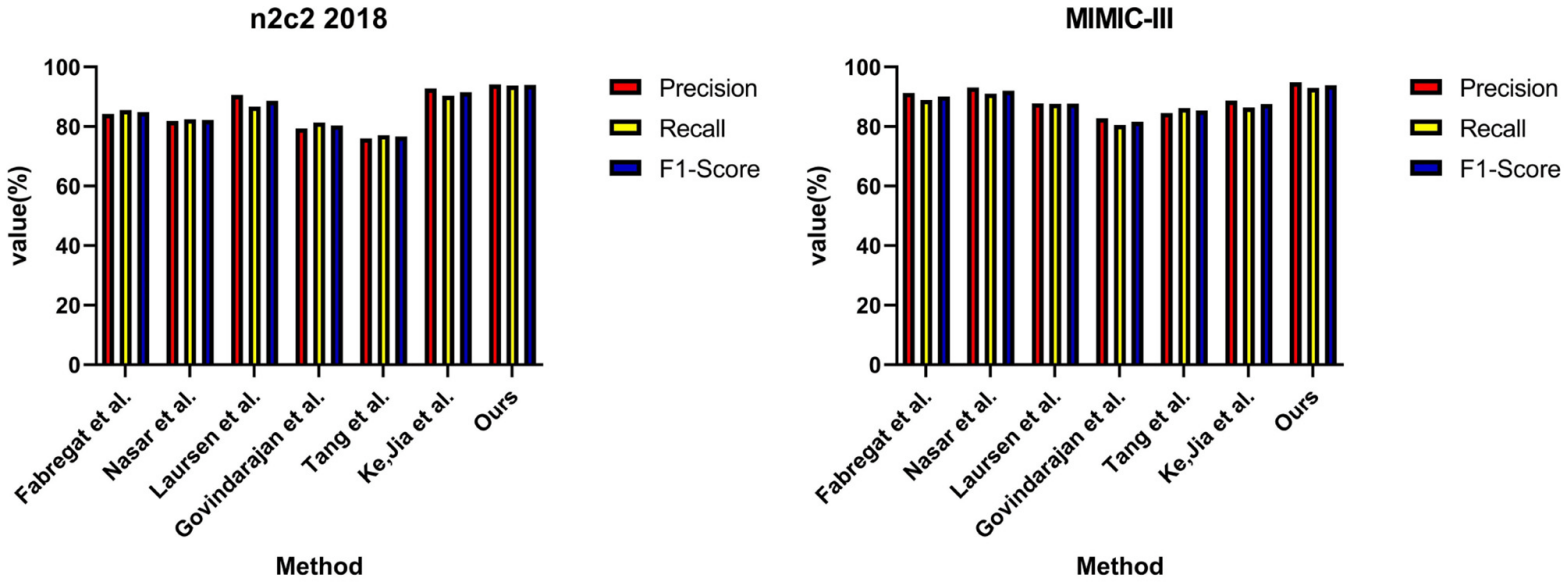
Comparative visualization of RE tasks for n2c2 2018 and MIMIC-III datasets.

Overall, from the quantitative results, it is evident that the EMLB model achieved significant improvements in the task through semantic representation learning and multi-task sharing. This validates the effectiveness of the proposed method. We have visually compared the results in Table 2, Figure 7.

From the experimental results in Table 3, we compared the performance of various models on the Clinical tempeval dataset and ShARe/CLEF dataset for the NER task. According to the data, we observed that the proposed EMLB model outperformed other models significantly on both datasets.

**Table 3:** Comprehensive evaluation based on precision, recall, and F1 score: different approaches for NER tasks on clinical tempeval and ShARe/CLEF datasets.

**Method**	**Dataset (for NER tasks**)					
	**Clinical tempeval (Bethard et al.**, **2016****)**			**ShARe/CLEF (Névéol et al.**, **2018****)**		
	**Precision/%**	**Recall/%**	**F1-score/%**	**Precision/%**	**Recall/%**	**F1-score/%**
Fabregat et al. (2023)	79.48	81.17	80.32	83.34	81.34	82.33
Nasar et al. (2021)	86.75	89.95	88.32	84.55	83.17	83.85
Laursen et al. (2023)	85.22	85.26	85.24	83.15	82.46	82.80
Govindarajan et al. (2023)	83.04	86.53	84.75	84.74	80.78	82.73
Tang et al. (2023)	78.38	76.14	77.24	90.44	88.21	89.31
Ke et al. (2023)	91.57	91.32	91.44	92.87	90.66	91.75
**Ours**	**94.22**	**95.05**	**94.63**	**95.39**	**94.34**	**94.86**

First, let's focus on the experimental results on the Clinical tempeval dataset. Our EMLB model achieved high precision (94.22%), recall (95.05%), and F1-Score (94.63%), indicating excellent overall performance in the NER task. Compared to other models, the EMLB model demonstrated a clear advantage in accuracy and recall. Next, we turn to the experimental results on the ShARe/CLEF dataset. Similarly, our EMLB model achieved remarkable performance on this dataset, with the highest precision, recall, and F1 value. This further validates the superior performance of the EMLB model in the NER task on the ShARe/CLEF dataset. Once again, the performance of our model outperforms other models on this dataset.

**Figure 8:**
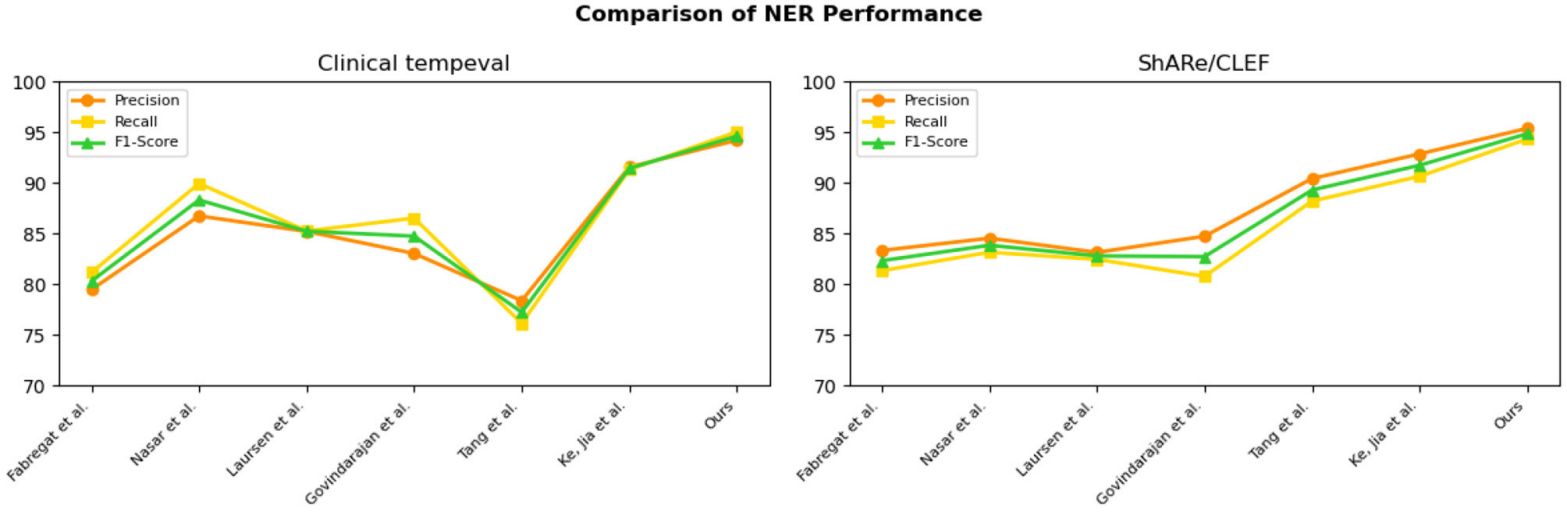
Comparative visualization of NER tasks for clinical tempeval and ShARe/CLEF datasets.

Overall, the experimental data clearly indicates that our proposed EMLB model achieves the best performance in the NER task on both the Clinical tempeval and ShARe/CLEF datasets. This fully demonstrates the effectiveness and excellence of the EMLB model in the NER task, providing an innovative solution for NER and in electronic medical records. We have visually compared the results in Table 3, Figure 8.

The experimental results on the Clinical tempeval dataset and ShARe/CLEF dataset for the RE task demonstrate that our proposed EMLB model has an advantage in this task. On the Clinical tempeval dataset, our model achieved 93.98% precision, 94.55% recall, and 94.26% F1-Score, significantly outperforming other models. This indicates that our model exhibits outstanding overall performance in the task. Similarly, on the ShARe/CLEF dataset, our model outperformed other models in precision, recall, and F1-Score, confirming its superiority in the task. These results illustrate that our proposed model performs well in NER and tasks in electronic medical records, contributing to further improvements in medical information extraction techniques and demonstrating the feasibility of our model in this field.

**Figure 9:**
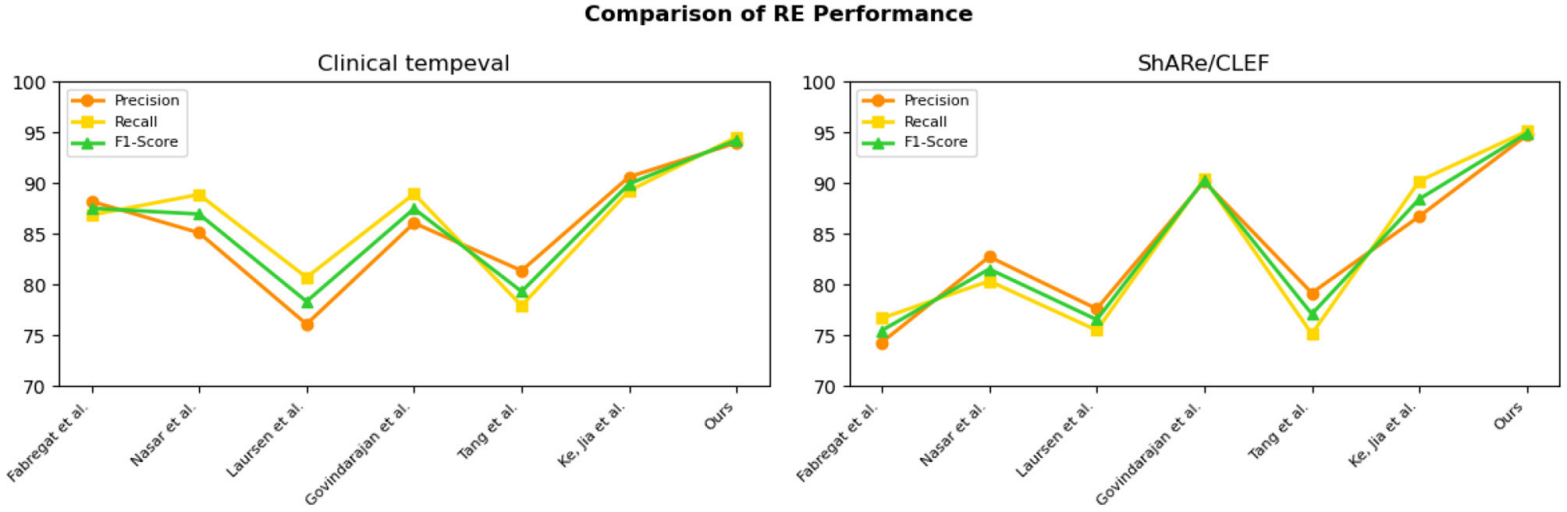
Comparative visualization of RE tasks for clinical tempeval and ShARe/CLEF datasets.

Following the performance comparison of the NER and RE tasks, we conducted further ablation experiments on the model to explore the influence of different factors on the performance of NER and RE. In the previous tables, we already compared the NER task performance of various models on different datasets in detail. By analyzing the results of the comparative experiments in Tables 5, 6, we can understand the performance of different models in NER and RE tasks and explore the impact of different factors on their performance. This will help us gain a comprehensive understanding of the strengths and limitations of the models and provide valuable guidance and references for future model design and applications. We have visually compared the results in Table 4, Figure 9.

**Table 4:** Comprehensive evaluation based on precision, recall, and F1 score: different approaches for RE tasks on clinical tempeval and ShARe/CLEF datasets.

**Method**	**Dataset (for RE tasks)**					
	**Clinical tempeval (Bethard et al.**, **2016****)**			**ShARe/CLEF (Névéol et al.**, **2018****)**		
	**Precision/%**	**Recall/%**	**F1-score/%**	**Precision/%**	**Recall/%**	**F1-score/%**
Fabregat et al. (2023)	88.21	86.87	87.53	74.31	76.69	75.48
Nasar et al. (2021)	85.12	88.89	86.96	82.75	80.36	81.54
Laursen et al. (2023)	76.11	80.72	78.35	77.62	75.51	76.55
Govindarajan et al. (2023)	86.08	88.95	87.49	90.18	90.42	90.30
Tang et al. (2023)	81.38	77.93	79.33	79.18	75.15	77.11
Ke et al. (2023)	90.63	89.26	89.94	86.74	90.23	88.45
**Ours**	**93.98**	**94.55**	**94.26**	**94.73**	**95.12**	**94.92**

Next, we will introduce the experimental results in Tables 5, 6 and their analysis in detail to gain a deeper understanding of the performance and performance differences of different modules.

**Table 5:** Comprehensive evaluation based on precision, recall, and F1 score: different modules for NER tasks on n2c2 2018, MIMIC-III, clinical tempeval, and ShARe/CLEF datasets.

**Module**	**Datasets (for NER tasks)**							
	**n2c2 2018 (Henry et al.**, **2020****)**		**MIMIC-III (Wang et al.**, **2020****)**		**Clinical tempeval (Bethard et al.**, **2016****)**		**ShARe/CLEF (Névéol et al.**, **2018****)**	
	**Precision/%**	**Recall/%**	**Precision/%**	**Recall/%**	**Precision/%**	**Recall/%**	**Precision/%**	**Recall/%**
Baseline	76.67	75.82	73.47	76.31	71.08	71.63	75.75	74.13
ete	83.84	83.02	86.31	83.45	80.62	83.92	79.81	80.48
MTL	86.01	83.47	87.79	84.33	88.46	88.93	87.92	89.93
ete MTL	93.33	92.39	94.51	93.93	93.28	94.64	94.38	95.76

Among them, ete means end-to-end module, MTL means multi-task learning module.

**Table 6:** Comprehensive evaluation based on precision, recall, and F1 score: different modules for RE tasks on n2c2 2018, MIMIC-III, clinical tempeval, and ShARe/CLEF datasets.

**Module**	**Datasets (for RE tasks)**							
	**n2c2 2018 (Henry et al.**, **2020****)**		**MIMIC-III (Wang et al.**, **2020****)**		**Clinical tempeval (Bethard et al.**, **2016****)**		**ShARe/CLEF (Névéol et al.**, **2018****)**	
	**Precision/%**	**Recall/%**	**Precision/%**	**Recall/%**	**Precision/%**	**Recall/%**	**Precision/%**	**Recall/%**
Baseline	83.71	80.15	78.61	81.24	81.53	81.72	80.03	82.34
ete	82.25	84.56	85.14	83.49	83.72	85.89	83.56	84.13
MTL	85.46	87.24	86.37	87.46	89.39	88.16	88.03	90.98
ete MTL	94.62	93.81	92.68	93.99	93.18	92.38	94.42	94.39

Among them, ete means end-to-end module, MTL means multi-task learning module.

In Table 5, we conducted a detailed analysis of the performance of the NER task on different datasets (n2c2 2018, MIMIC-III, Clinical tempeval, and ShARe/CLEF). Firstly, we focused on the experimental results on the n2c2 2018 dataset. The baseline module showed moderate performance with a Precision of 76.67% and Recall of 75.82%. However, with the introduction of the ETE module, the performance significantly improved, achieving a Precision of 83.84% and Recall of 83.02%. Further application of the Multi-Task Learning (MTL) module resulted in further improvements, with Precision reaching 86.01% and Recall at 83.47%. These findings demonstrate the significant performance enhancement of the ETE and MTL modules on the NER task in the n2c2 2018 dataset.

Moving on to the experimental results on the MIMIC-III dataset, similar to the n2c2 2018 dataset, the baseline module exhibited moderate performance with a Precision of 73.47% and Recall of 76.31%. The ETE module improved the Precision to 86.31% and Recall to 83.45%. With the MTL module, the Precision further improved to 87.79%, while the Recall reached 84.33%. Once again, these results confirm the effectiveness of the ETE and MTL modules, showing significant performance improvements on the NER task in the MIMIC-III dataset.

For the Clinical tempeval dataset, the baseline module showed relatively lower performance, with a Precision of 71.08% and Recall of 71.63%. However, under the guidance of the ETE module, the Precision and Recall increased to 80.62 and 83.92%, respectively. The MTL module further enhanced the performance, achieving a Precision of 88.46% and Recall of 88.93%. These results again demonstrate the effectiveness of the ETE and MTL modules in improving performance on the NER task in different datasets.

Lastly, we focused on the experimental results on the ShARe/CLEF dataset. The baseline module exhibited a Precision of 75.75% and Recall of 74.13%, showing moderate performance. With the ETE module, the Precision and Recall increased to 79.81 and 80.48%, respectively. The MTL module further improved the Precision to 87.92% and Recall to 89.93%. These results again confirm the superiority of the ETE and MTL modules on the NER task.

**Figure 10:**
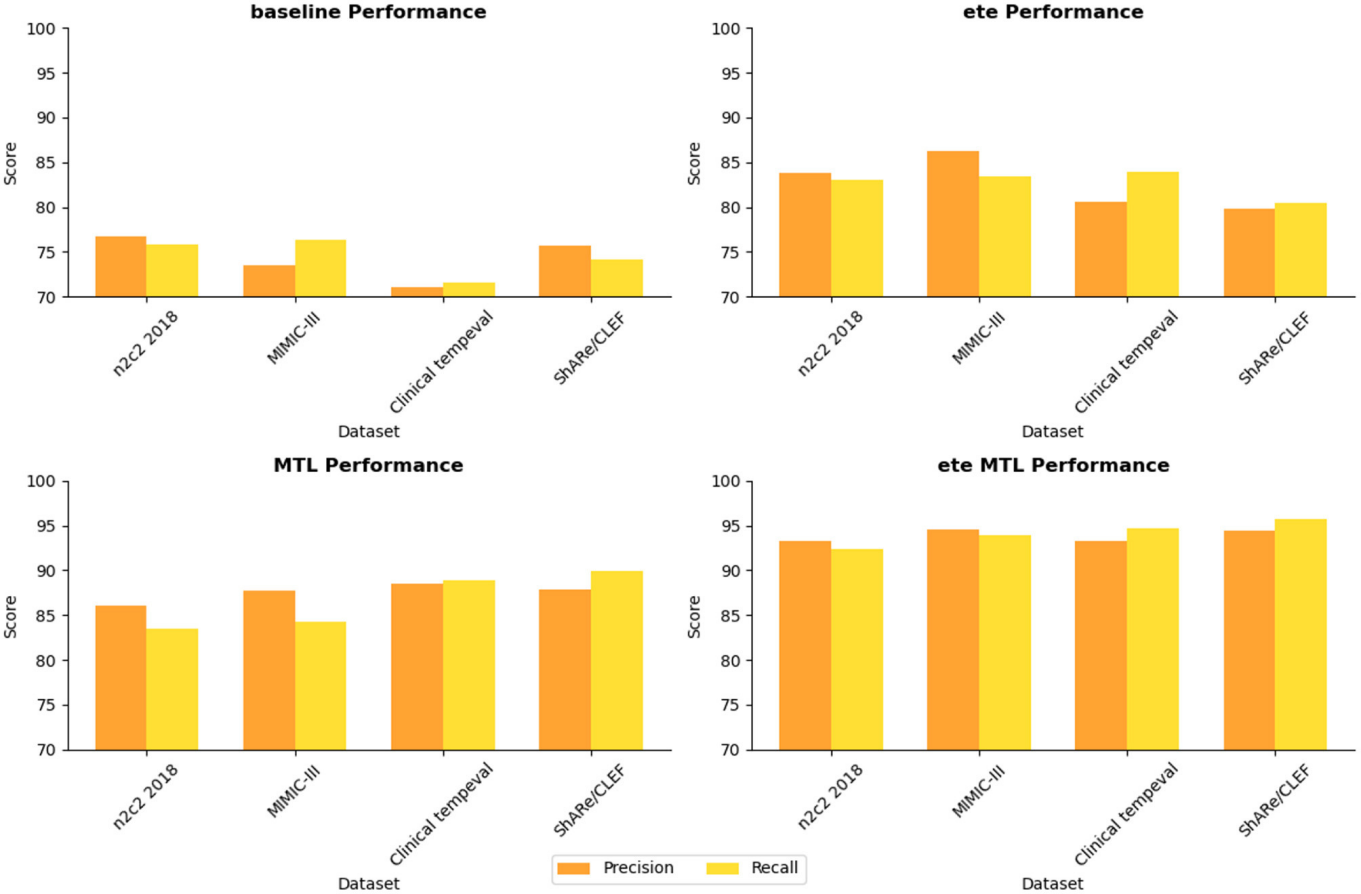
Visualization of NER task performance comparison based on different modules.

The ETE and MTL modules demonstrated significant performance improvements on the NER task across different datasets. Especially in the ETE MTL module, the simultaneous use of ETE and MTL achieved the best performance, with very high Precision and Recall. Thus, our experimental results indicate that the introduction of Multi-Task Learning is crucial for NER in electronic health record texts and provides an effective solution for the NER task. We have visualized the results in Table 5 for comparison in Figure 10.

In Table 6, we present a performance comparison and analysis of the RE task on different datasets (n2c2 2018, MIMIC-III, Clinical tempeval, and ShARe/CLEF). Firstly, we focus on the experimental results on the n2c2 2018 dataset. The baseline module showed good performance on the task, achieving a Precision of 83.71% and Recall of 80.15%. However, with the introduction of the ETE module, there was a slight decrease in Precision to 82.25% and an increase in Recall to 84.56%. Under the guidance of the MTL module, the performance improved again, reaching a Precision of 85.46% and Recall of 87.24%. Remarkably, the ETE MTL module, combining ETE and MTL modules, achieved the best performance with a Precision of 94.62% and Recall of 93.81%, far surpassing other modules on the n2c2 2018 dataset.

Next, we turn to the experimental results on the MIMIC-III dataset. The baseline module demonstrated good performance with a Precision of 78.61% and Recall of 81.24%. After incorporating the ETE module, Precision and Recall increased to 85.14 and 83.49%, respectively. With the MTL module, Precision further improved to 86.37%, and Recall to 87.46%. In the ETE MTL module, Precision was 92.68%, and Recall was 93.99%. These results once again validate the performance improvement of the ETE and MTL modules on the task in different datasets.

For the Clinical tempeval dataset, the baseline module showed relatively higher performance with a Precision of 81.53% and Recall of 81.72%. With the ETE module, Precision and Recall increased to 83.72 and 85.89%, respectively. Under the MTL module, Precision further improved to 89.39%, and Recall was 88.16%. In the ETE MTL module, Precision reached 93.18%, and Recall was 92.38%. These results again confirm the superiority of the ETE and MTL modules on the task in different datasets.

Finally, we focus on the experimental results on the ShARe/CLEF dataset. The baseline module demonstrated moderate performance with a Precision of 80.03% and Recall of 82.34%. After applying the ETE module, Precision and Recall increased to 83.56 and 84.13%, respectively. Under the MTL module, Precision further improved to 88.03%, and Recall was 90.98%. In the ETE MTL module, Precision reached 94.42%, and Recall was 94.39%. These results indicate significant performance improvements of the ETE and MTL modules on the task in the ShARe/CLEF dataset.

**Figure 11:**
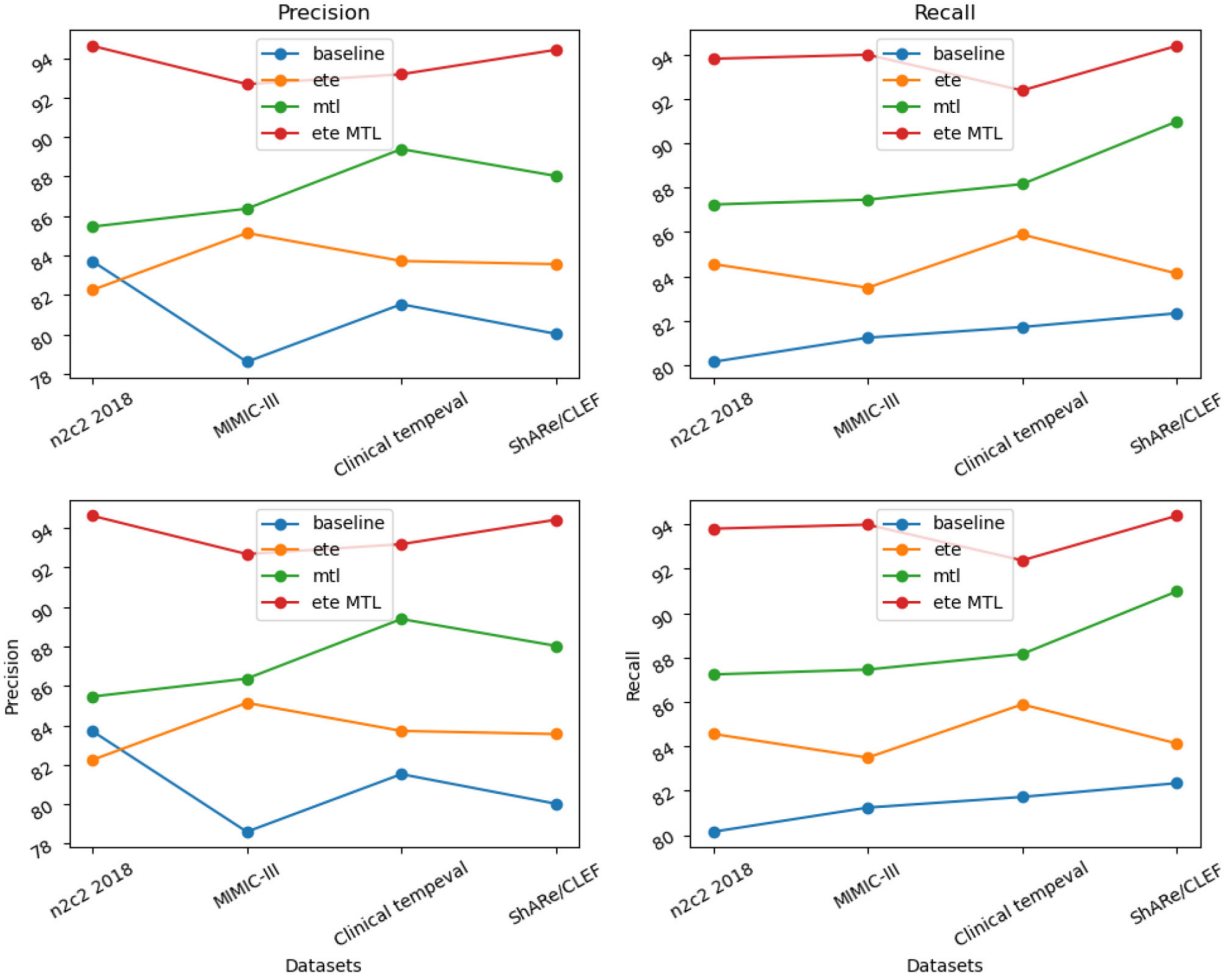
Visualization of RE task performance comparison based on different modules.

Overall, the ETE and MTL modules exhibited significant performance improvements on the task across different datasets. Especially in the ETE MTL module, the simultaneous use of ETE and MTL achieved the best performance with very high Precision and Recall. Thus, our experimental results show that Multi-Task Learning and the combination of ETE and MTL modules are of great significance for electronic health record, providing an effective solution for the RE task. These results further validate the excellent performance of our proposed method in NER and tasks on electronic health records, providing valuable insights for information extraction technology in the medical domain. We have visualized the results in Table 6 for comparison in Figure 11.

## 5. Discussion

Our research aims to explore the integration of natural and deep artificial cognitive models in medical image processing, focusing on the NER and RE tasks in EMR texts using BERT-based methods. In this section, we will discuss the results of our study, with a specific emphasis on the impact of integrating natural and artificial cognitive systems in medical image processing and the performance of the BERT-based approach in analyzing EMR texts.

Regarding the processing of EMR texts, our research employs BERT-based methods for NER and. The experimental results demonstrate that the BERT model performs exceptionally well in the NER and RE tasks of EMR. Its pre-training ability and bidirectional encoder representations enable the model to better comprehend the semantic and contextual information of medical texts, leading to more accurate and comprehensive entity recognition and. This provides robust support for the intelligent processing of EMR and medical information mining. Firstly, our model combines BERT's pre-training capability, allowing it to learn rich semantic representations from vast amounts of EMR data. This enhanced understanding of medical terms and specialized vocabulary improves the accurate identification and extraction of domain-specific entities, empowering healthcare professionals to swiftly and accurately locate crucial information in EMR texts, saving time in data retrieval and organization, and enhancing work efficiency. Secondly, our model adopts an end-to-end architecture that fully leverages BERT's underlying representations and utilizes multi-task learning to facilitate knowledge sharing between NER and RE tasks, reducing the limitations of independent steps and risks of error propagation in traditional approaches. This contributes to more comprehensive and precise entity relationship extraction in the medical domain, helping healthcare professionals better understand the connections and interactions between different entities. For instance, by accurately extracting the relationship between medications and diseases, doctors can gain a better understanding of treatment effectiveness, optimize medication plans, and provide more personalized and effective care for patients.

However, despite the many advantages demonstrated by the integration of natural and artificial cognitive systems in medical image processing and EMR handling, some challenges remain. For instance, different types of medical images and diverse EMR texts pose specific requirements for data input and processing in integrated systems. Additionally, the interpretability and explainability of the models are crucial, especially in medical decision-making scenarios where understanding the model's decision process is essential. Looking ahead, further research and improvements in the integration of natural and deep artificial cognitive models, especially when dealing with more complex, multimodal data, and diversified tasks, are needed. Additionally, for BERT-based methods, exploring larger and more diverse pre-training corpora can enhance the model's generalization and adaptability. Furthermore, combining the integration of natural and artificial cognitive systems will bring forth more application scenarios and innovations in medical image processing and EMR handling, providing comprehensive support for the intelligent development of the medical field and better healthcare services for patients.

## 6. Conclusion

The aim of this research is to explore the integration of natural and deep artificial cognitive models in medical image processing, focusing on the NER and RE tasks in EMR using BERT-based methods. We propose an integrated framework that combines natural language processing with deep learning for medical image analysis. This framework utilizes the BERT model for NER and RE tasks on EMR as prior knowledge for subsequent medical image analysis. The BERT-based end-to-end framework, which integrates NER and RE tasks into a unified model and applies the concept of multi-task learning, achieves significant performance improvements. Through comprehensive experiments on different datasets, we validate the effectiveness and superiority of the proposed model. The research results demonstrate that incorporating semantic information can significantly enhance the understanding and analysis of medical images compared to using solely computer vision methods, providing valuable insights for medical intelligence research and applications.

However, the NER and RE tasks in EMR still pose challenges and remain open issues. With the continuous growth of medical information, EMR text data becomes more extensive and complex, demanding higher model performance and efficiency. In future research, we will continue to explore more efficient and accurate model designs to cope with the increasing volume of medical data. Especially for extracting specific medical entities and relations in certain domains, we can investigate more specialized and customized model designs to improve the model's understanding and application of domain-specific terminologies and knowledge. Additionally, for cross-lingual and cross-domain information extraction problems, we can further study techniques such as transfer learning and domain adaptation to expand the model's applicability in different domains and languages. The research outcomes of this paper make significant contributions to the field of NER and in electronic medical records. In future research, we will continue to advance medical information extraction technology, providing more efficient and accurate solutions for medical intelligent applications, thereby making even greater contributions to the development and improvement of the medical field.

## Data availability statement

The original contributions presented in the study are included in the article/supplementary material, further inquiries can be directed to the corresponding author.

## Author contributions

BG: Conceptualization, Data curation, Formal analysis, Funding acquisition, Methodology, Software, Validation, Writing—original draft. HL: Data curation, Funding acquisition, Investigation, Project administration, Writing—original draft. LN: Formal analysis, Investigation, Methodology, Resources, Writing—original draft.
